# Monitor for lactate in perspiration

**DOI:** 10.1186/s12576-021-00811-3

**Published:** 2021-08-26

**Authors:** Ting-Ting Luo, Zhong-Hai Sun, Chu-Xin Li, Jin-Lian Feng, Zhao-Xiu Xiao, Wei-Dong Li

**Affiliations:** 1 School of Nursing, Institute of Health, Guangdong Pharmaceutical University, Guangzhou, China; 2 Department of Surgery, The First Affiliated Hospital of Guangdong Pharmaceutical University, Guangzhou, China; 3 Department of Thoracic Surgery, The First Affiliated Hospital of Guangzhou Medical University, Guangzhou, China; 4 Department of Cardiac Surgery, The First Affiliated Hospital of Guangzhou Medical University, Guangzhou, China

**Keywords:** Sweat metabolism, Ischemia, Soft tissues, Lactate

## Abstract

Sweat is a noninvasive biological fluid on the surface of human skin and has attracted increasing attention as a diagnostic specimen for disease and biomarker detection. Sweat metabolite quantification is possible due to progress in sweat analysis techniques; nevertheless, the role of sweat monitoring in energy metabolism, physiological or pathological state assessment, health status assessment, and the development and outcome of metabolism-related diseases remains unclear. This review provides a comprehensive overview of the literature on human sweat lactate concentration. The first, second, and third sections of this review present an introduction of sweat lactate, methods for the collection and storage of sweat lactate samples, and methods of detection and analysis of sweat lactate, respectively. The fourth section elaborates upon the current state of clinical application of sweat lactate monitoring and its prospects for health surveillance. The last section focuses on the challenges and future directions of this novel technology for detecting lactate in sweat.

## Background

The mainstream of modern medicine has assessed the physiological state of the human body by measuring analytes in body fluids such as blood and urine. However, these measurement modalities must be handled by professionals, and obtaining body fluids may require the use of invasive procedures. Analyzing the composition of sweat is innovative and convenient for human body status monitoring. Humans have 4 million exocrine glands. Sweat secretion is directly involved in body temperature and metabolism regulation; moreover, sweat is easily collected and may contain noninvasive, health-related biomarkers. Perspiration has long been used to screen for cystic fibrosis (CF) [1–3]. It is important to explore the feasibility of using biomarkers in sweat for early diabetic leg ulcer occurrence prediction, disease diagnosis, drug metabolism detection [4–6]. Saliva, sweat, tears, and other biological fluids are obtained in a noninvasive way, and these biomarkers are used to track the changes in metabolites in the body, thereby facilitating the noninvasive evaluation of physiological status and improving health [7]. Precise noninvasive biomarker acquisition is the holy grail of biomedicine [8]. Human eccrine gland transpiration is a liquid rich in biomarkers, which has attracted attention over the last 100 years.

Yasu Kuno’s publication of *The Physiology of Human Perspiration* in 1934 [9] triggered an unending interest in sweat lactate exploration. With the recent development of portable sensors, sweat exploration has reached a new peak. Professor Kuno’s research provided the basis for our quantification of metabolites in sweat [9]. However, Heikenfeld [7] considered that the huge cost of portable technology in biological fluid monitoring does not favor investment in the study of biofluid physiology. In particular, the basic biochemistry of metabolites such as sweat lactate has been the focus of many studies; moreover, research on sweat composition and the mechanism of sweat lactate detection has been stagnant, although many scholars have studied sweat lactate. However, it is unclear whether sweat lactate concentration can reflect the human physiological state. Human sweat technology is a new technology that attempts to use lactate and other metabolites in sweat to noninvasively monitor the human physiological state. This noninvasive monitoring encompasses personal health surveillance, clinical analysis and diagnosis, nutritional status surveillance, as well as athletic and military medicine [10–14].

Sweat originates primarily from sweat glands. This review deals mainly with sweat secreted by the eccrine glands, because the quantifiable amount of sweat is small relative to that of other biological fluids, and the eccrine sweat glands are more numerous (2–4 million) than apocrine sweat glands [1]. Compared to apocrine glands, eccrine glands contains fewer bacteria and oils than apocrine glands, making it possible to quantitatively detect lactate in sweat [7]. Sweat plays a major role in regulating the temperature of the human body. Water constitutes 99% of sweat, which contains components such as lactate, sodium, chloride, and urea. Lactate is one of the major metabolites that can be monitored in sweat. The mechanism of sweat production is complex, and the sources of various sweat components remain unclear. Sweat glands produce a small amount of lactate under physiologic conditions; nonetheless, some factors may induce anaerobic metabolism, resulting in increased lactate production during glycolysis [2]. Many scholars assume that sweat lactate level increase is related to sweat gland fatigue [15, 16]. By studying the inducing factors leading to sweat lactate level increase, it may be possible to monitor the physiological status. In fact, it has been shown that athlete sweat lactate concentration increases during training and skin and soft tissue compression due to sweat gland fatigue, and returns to normal values after removing the inducing factor.

This review focuses on the development and application of human sweat technology research using sweat lactate. With advances in science and technology, our understanding of the biomedical applications of sweat lactate continues to increase. As a noninvasive biomarker, sweat lactate monitoring can fundamentally change patient monitoring and treatment. Consequently, there has been a surge in academic research on sweat lactate and the commercial use of sweat lactate-based sensors, which may be used in the future to track athlete endurance during exercise, evaluate skin and soft tissue status, and ensure personal identification [11–13]. However, the understanding of basic and complex sweat physiology is insufficient, and therefore, the application of sweat in health status monitoring is somewhat limited. This paper reviews the mechanism of sweat lactate detection and its applications; the rapid development of sweat lactate detection methods is promising for continuous health and physiological monitoring. Moreover, we reviewed methods of sweat sample collection and storage, methods of sweat lactate detection and analysis, and the objective of sweat lactate monitoring. Finally, we discussed the challenges involved in developing these types of lactate monitoring techniques, as well as orientations on strategies to overcome these challenges in the future.

## Natural method of sweat collection for lactate analysis

**Fig. 1:**
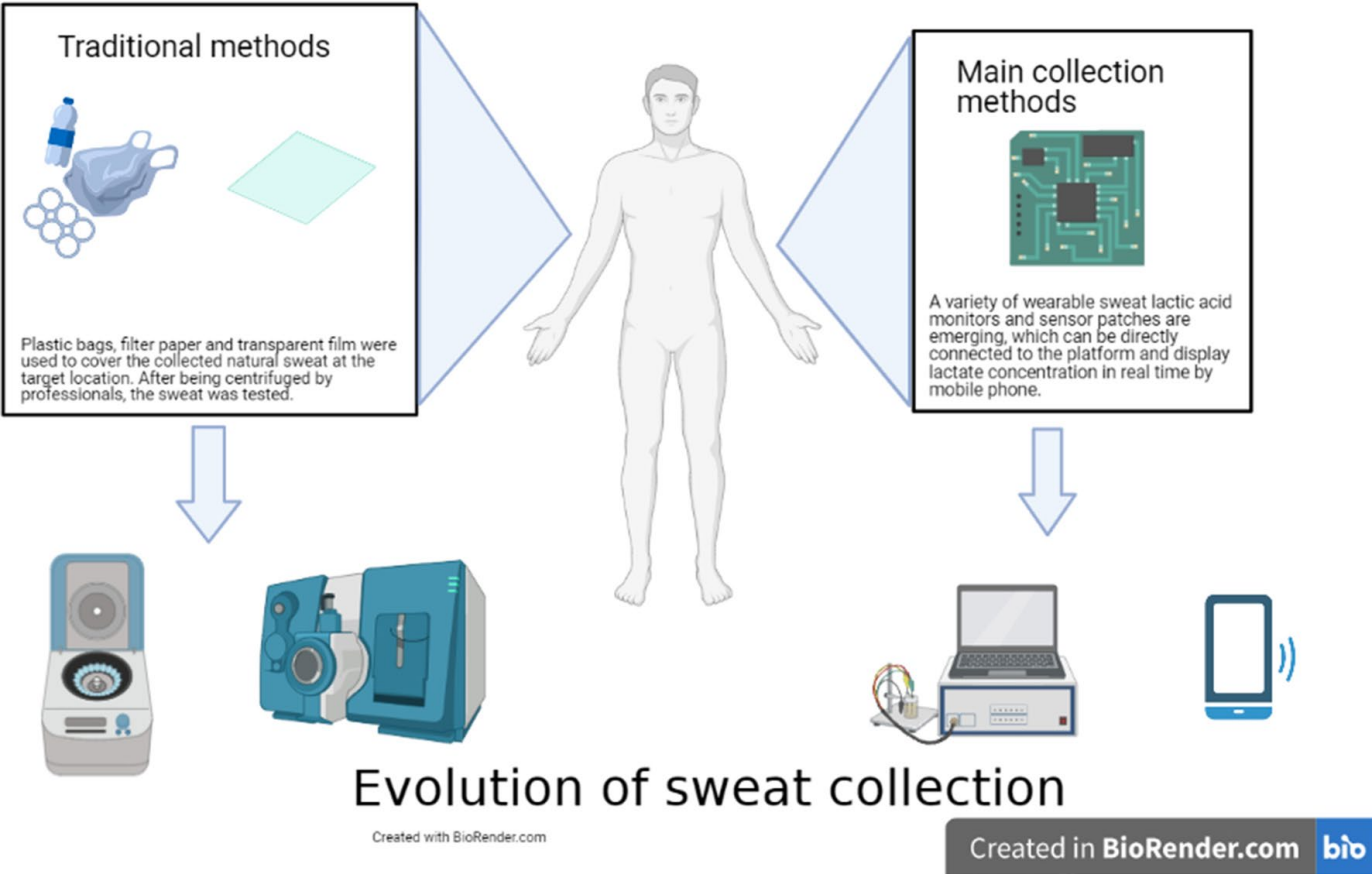
Evolution of sweat collection Methods: **A** traditional plastic bags and filter papers are used to collect sweat, which is then tested in the laboratory. **B** Sensors collect sweat and detect sweat lactate concentration, which is then exported using mobile transmission devices

Traditionally, sweat is collected using textiles and plastic bags, and treated and analyzed by professionals in the laboratory [2, 15]. In addition, to avoid sweat evaporation, a transparent film with little irritation to the skin and a waterproof polypropylene film are used to cover filter paper pads (Whatman Chromatography, Whatman paper, Maidstone, UK) or paraffin oil film [2, 15]. A variety of wearable sweat lactate monitors and sensor patches have been developed, including wearable monitors with hydrophobicity, skin protection, and good electrical conductivity, as well as textile sensor patches, which can simultaneously detect glucose, lactate, ascorbic acid, Na+, and other substances in sweat [8, 10, 17]. Wearable sweat lactate monitors can be used to continuously and stably monitor sweat lactate and are advantageous in terms of long-term exposure to acidic conditions as well as sweat evaporation and contamination prevention [10, 18]. The emerging wave of technology favors the noninvasive real-time sweat lactate biomarker monitoring of the human physiological state; furthermore, noninvasive human sweat analyte measurement is used as an alternative to blood analyte measurement. Although parameters such as glucose, ascorbic acid, Na+ are valuable in assessing the body’s physiologic state, they do not seem to change dynamically with changes in metabolic and physiologic processes. Figure 1 shows the evolution of sweat collection methods.

## Non-natural method of sweat collection for lactate analysis

Depending on eccrine gland location in the body, the density of eccrine glands varies from dozens to hundreds of glands per square centimeter [7]. Thus, the total amount of sweat secreted per minute may vary from 1 to 1000 nL min^−1^ cm^−2^ [7]. Before the advent of wearable sensors, the sweat collection process took 20 min to 7 h per sample [15, 19, 20]; hence, pilocarpine-induced electrical stimulation was used to increase sweat productivity and shorten sweat collection time. The abovementioned stimulation procedure involves the use potential difference stimulation via positive and negative electrodes to increase the sweat flow rate of eccrine glands. A filter paper infiltrated with 1% pilocarpine solution is placed between the forearm skin and the positive electrode, and magnesium sulfate electrolyte solution (0.05 mL) is added to the negative electrode to obtain a 0.4–0.5-mA current within 10 min. Thereafter, the filter paper is placed on the skin, covered with paraffin film, and fixed with a tape. Sweat is collected after 20 min following the reduction of sweat evaporation and simultaneous sweat absorption [19]. Some researchers believe that sweat collection after exercise- and pilocarpine-induced stimulation is more reproducible than natural sweat collection [19]. However, we believe that non-natural methods of sweat collection can reduce the possibility of sweat evaporation and induce chemical changes in endogenous analytes. Furthermore, there is a risk of burn injuries, and other complications; hence, the safety and feasibility of these non-natural methods require verification. With the recent increase in the use of portable sensors, sweat collection using ways that can be potentially harmful to participants is not advisable.

## Storage of sweat samples for lactate analysis

After conventional sweat collection, it is stored at − 80 °C [15, 20] and thereafter removed for analysis when required. There is no standardized procedure for sweat storage, and the sweat storage method used can potentially induce a change in sweat composition. Moreover, the sweat composition may change over time and the sweat components may evaporate, resulting in an increase in sweat electrolyte concentration. To the best of our knowledge, no study has explored the optimum storage temperature of sweat for lactate conservation. With the development of portable sweat sensors, real-time sweat composition results can be produced without having to undergo the abovementioned tedious steps.

## Biochemical analysis of sweat lactate

Sweat lactate can be detected using laboratory analysis or wearable sensors. The former involves the collection, processing, and sending of sweat samples to the laboratory for professional testing, whereas the latter involves sweat collection via wearable devices, which can directly display important information about the individual’s physiology. Presently, the main methods of real-time health and fitness surveillance for detecting sweat lactate are the lactate dehydrogenase and electrochemical detection methods [20–28]. These methods can be used to evaluate changes in sweat lactate concentration related to anaerobic threshold in endurance exercise, and to monitor lactate acidosis in intensive care patients [21]. Continuous real-time simultaneous detection of glucose, lactate, sodium, and potassium can be realized; sweat lactate concentration can be tracked in real time, its fluctuation monitored, and the electrophysiological and chemical signals measured simultaneously [29–34]. Portable sensors are used to monitor the sweat function of cyclists performing long-distance fitness cycling in both dry and outdoor conditions. Monitored sweat parameters include transpiration rate, total transpiration, pH, and chloride and lactate concentrations [29, 30].

Mass spectrometry is the traditional method of sweat lactate measurement Sweat lactate can be quantified by absorbance measurement, matrix-assisted laser desorption/ionization mass spectrometry (MALDI-MS), ultra-high-performance supercritical fluid chromatography–mass spectrometry, multidimensional spectroscopy, and high-performance liquid chromatography [2, 15, 20, 31]. The abovementioned sweat lactate detection methods are usually used for artificially collected sweat after laboratory treatment. However, it is crucial to help researchers determine the concentration of sweat compounds by early sweat exploration.

In addition, a new nuclear magnetic resonance technique was used to collect sweat from different parts of the human body, and the minimum surface area required for sampling ranged from 50 to 100 cm [19]. The most common organic compounds found in healthy human sweat were lactate, glycerol, pyruvate, and serine; 34 different metabolites were quantified [19]. This new exploration technique uses composite materials to improve sweat lactate detection. Wearable sensor systems are composed of organic electrochemical transistors, sensors with a new silicon material, and nanostructured ZnO films combined with Laday biosensors to support a wide range of personalized diagnostic and physiological monitoring applications [31–34]. A lower detection limit and a wider dynamic range of lactate biomarker detection have been quantified. (Details of specific methods are found in Table 1).

**Table 1:** Summary of main sweat lactate monitoring methods and a comparison of their advantages

Detection method		Resource	Range of lactate concentration detection	Test duration	Advantages	Application
Lactate dehydrogenase method		Wearable sensor [21, 27, 28]	0.05 mM 5–100 mM 20–60 mM	5 min [21]	Simple and economical	Assess changes in lactic acid associated with anaerobic threshold during endurance training and monitor lactic acidosis in critical care patients
		Lab [2, 15, 20]	Measure the range depending on the kit	12.5 min	Economical	Potential applications of soft tissue skin conditions
Electrochemical detection	Voltage method [27]	Wearable sensor	20–60 mM [27]	Real time	High sensitivity, low cost, and small size	Simple, non-invasive monitoring of sweat biomarkers on a regular daily basis
	Resistance method [10]		3–100 mM	2–3 min	It is more stable and lasts 6 months	For future non-invasive real-time clinical studies and sports medicine
	Amperometric detection [22–24, 28]		51 μM 0.01–18.4 mM	Real time	High sensitivity, excellent selective for lactate	Devices on a chip that have the potential for a wide variety of applications in biosensors, bioelectronics, and labs
	Colorimetric chemistry techniques [25, 29, 30]		0–20 mM 1–100 mM	Real time	Lighter, cheaper, and smaller	Fitness, cycling, and long distance cycling races in both dry and outdoor conditions
MALDI-MS [2, 15, 20]		Lab	20.6–72.9 mM	Delay	One of the most important monitoring methods	The primary method for sweat lactate detection
MRI [19]		MRI room	–	Delay in detection results	Thirty-four different metabolites were detected quantitatively	Insufficient data
Composites [31–34]		Wearable sensor	1–180 mM [31] 1–100 mM [32]	Real time	Finding multiple target analytes [32]	Diagnostic and physiological surveillance applications

*Lab* laboratory, *MRI* magnetic resonance imaging, *MALDI-MS* matrix-assisted laser desorption/ionization mass spectrometry

## Lactate concentration

Sweat glands secrete a small amount of lactate under physiological conditions, and the human sweat lactate concentration is approximately 16–30 mM [16]. Different body parts have different sweat lactate concentrations. A previous study included 49 healthy participants who performed moderate-intensity exercise without acidosis occurrence. After cycling for 20 min at an ambient temperature of 42 °C and 20% relative humidity, the lactate concentrations in sweat collected from the backs of girls and boys were 23.6 ± 1.02 mM and 21.2 ± 1.7 mM [35], respectively. Moreover, the lactate concentration in sweat collected from the arms of men was approximately 15 mM [36]. There was a difference between the lactate concentrations in sweat collected from the sacral and coccygeal regions [2, 15, 20]; however, due to difference in the volume of sweat secretion in the different parts, the difference in concentration was small [2, 16]. The reason for the difference in lactate concentration in different locations has not been studied; nonetheless, it may be related to the secretion of local soft tissue glycogen decomposition products.

**Fig. 2:**
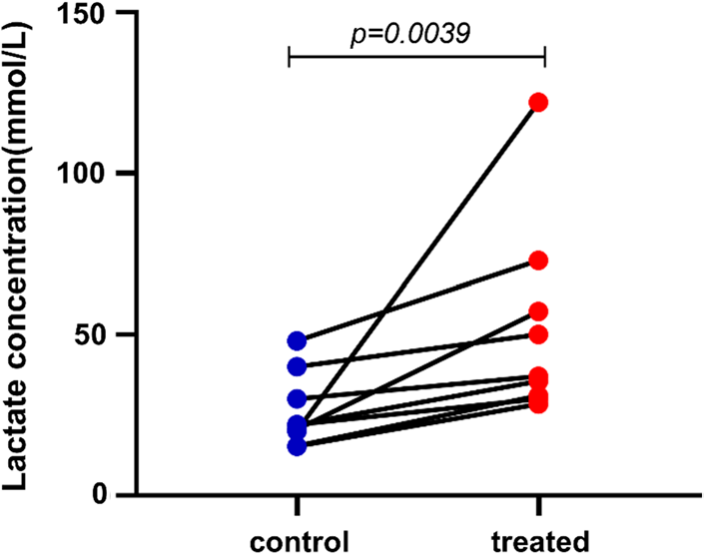
Changes of lactate concentration in the sweat of sacrum skin after different loading

Muscle fatigue and some pathological conditions such as soft tissue hypoxia and cystic fibrosis can increase sweat lactate concentration [2, 5, 16]. A previous study used wearable chemical sensors to monitor sweat lactate concentrations in cyclists during exercise, and found that the values exceeded 100 mM during exercise, which was over 10 times higher than blood lactate concentrations [31]. This is probably due to exercise-induced muscle fatigue. Glycolysis occurs in the human body, leading to an increase in sweat lactate concentration. The findings of the present review on sweat lactate physiology suggest that hypoxia leads to increased glycolysis, which is consistent with the findings of Derbyshire et al. who stated that walkers (aerobic) have the lowest concentration of sweat lactate, followed by footballers (anaerobic); tennis players (anaerobic) have the highest concentration of sweat lactate [16]. In addition, when soft tissue was subjected to different degrees of pressure loading as well as local tissue ischemia and hypoxia, the local sweat lactate concentration increased by 24% to 70% of the baseline concentration [15], and the lactate concentration ranged between 20 and 122 mM [2, 15, 20] (Fig. 2).

## Potential medical application of sweat lactate

### Early pressure ulcer prediction

Pressure injury (PI) is defined by the international consensus on pressure ulcers/injuries as “local damage to the skin and/or underlying soft tissue, caused by long-term mechanical load due to pressure or a combination of pressure and shear force, usually under a bony prominence” [36]. Globally, PI is one of the most common adverse events among hospitalized patients [37]. PIs not only increase national health care spending and the burden on medical staff, but also lead to unnecessary patient suffering.

PI results from two injury mechanisms: short-term pressure-induced cell injury and prolonged (several hours) biomechanical pressure-induced capillary and lymphatic vessel closure, resulting in ischemia–reperfusion injury [38]. Any of the above mechanisms will hinder the transport of important nutrients to cells, thereby causing a shift from aerobic to anaerobic cellular metabolism. The accumulation of anaerobic metabolites, such as lactate and pyruvate, decreases local soft tissue pH. Apart from the abovementioned two principal mechanisms, there is a limited risk assessment of the human cause of PI in the clinical setting. This review focuses on hospital-acquired PI. The prevalence of PI is 8.4%; the most frequent PI is pressure ulcers (stage 1 PI), accounting for approximately 43.5% of PIs [37]. Hibbs et al. found that 95% of pressure sores are preventable [39]; hence, early pressure sore detection and prevention is crucial. Currently, PI risk assessment is primarily based on PI risk assessment scales such as the Norton, Braden, and Waterlow scales [40–42]. Over the last 30 years, medical workers, particularly nurses, have used these scales to identify people at risk for PI development. These scales are primarily the result of expert advice and literature review. However, no study has shown the efficacy of these scales in PI prediction [43]. The choice of PI prevention strategy depends primarily on the naked eye examination, palpation, and rating ability of clinical nurses [36]. Moreover, nurses use their clinical experience to evaluate patients and provide corresponding PI preventive care; this method of evaluation cannot furnish nurses with information pertaining to the physiologic state of the subcutaneous tissue. Therefore, in clinical practice, there is an urgent need for an objective and timely monitoring strategy for tissue ischemia, hypoxia, and muscle fatigue, to facilitate early pressure sore detection and trigger a timely implementation of PI preventive measures by clinical staff. This would reduce unnecessary PI-induced pain and medical expenses incurred by patients.

Sweat lactate, an anaerobic metabolite, can become a noninvasive biomarker as an early indicator of ischemic tissue damage, as first reported by Van Heyningen et al. in 1952 [2]. Pressure ischemia can lead to undesirable clinical consequences such as PI development; thus, it is essential to prevent or diagnose pressure ischemia as early as possible before symptoms develop. In 1993, Polliack et al. [44] measured the concentrations of lactate, chloride, and uric acid in sweat produced by ischemic tissue in the sacral region during wheelchair sitting and lying down, after measuring the same parameters in soft tissues of healthy participants under different pressure loads; however, after reperfusion, the concentrations of these metabolites returned to pre-pressure values, suggesting that some metabolites could be used to indicate soft tissue injury. In 1997, Polliack et al. [45] studied 11 individuals who participated in rehabilitation, and found that the baseline sweat lactate concentration at the sacral compression area increased by 39% after removing the load pressure. Recently, Soetens conducted a study with a small sample of healthy people who underwent continuous or intermittent pressure application on the sacral region; they showed that the sweat lactate concentration of the sacral skin was higher than that of the tissues without applied pressure, and the increase in metabolite lactate content in the sweat ranged from 30 to 39% [15]. After the pressure was removed and the local tissue was reperfused, the sweat lactate concentration returned to the baseline value [15, 44, 45]; these results were similar to those of other studies.

Based on the known PI mechanism, Mansfield et al. [46] suggested that biomarker monitoring is the most promising strategy for preventing PI in the future. A study of healthy individuals highlighted that biomarkers such as sweat lactate, total sebum protein, interleukin (IL)-1α, and C-reactive protein can be used to monitor skin metabolic activity and local soft tissue ischemia and hypoxia; moreover, these potential biomarkers can predict the occurrence of PI [15, 47–49]. Although these biomarkers can detect the degree of stress-induced tissue damage, samples are collected by professionals via an invasive procedure. In fact, it is suggested that skin sebum IL-1α and sweat lactate may be used as potential noninvasive biomarkers to predict the occurrence of pressure ulcers [15, 48]. The sweat gland, as a metabolic unit, reflects its oxygenation state by increasing lactate production; hence, sweat lactate concentration can reflect local tissue activity and response to pressure. As a potential means to monitor skin and soft tissue health under pressure, metabolites may interfere with PI prevention over time. However, no large-scale studies have used sweat metabolites as early indicators of PI occurrence. Given the current understanding of the PI mechanism and ethical requirements, the assumption the sweat metabolites could be early indicators of PU occurrence requires further verification. To demonstrate the relationship between increased metabolite concentration and soft tissue ischemic blood oxygen, some scholars have suggested that if ethical approval is obtained, the concentration of metabolites in grade I PI can be measured [15].

### Disease diagnosis

Sweat lactate concentration monitoring technology is a relatively common method for sweat detection that is widely used by researchers; there is an increasing number of methods used to monitor and apply sweat lactate concentration in the field of medical biochemistry. Human sweat, as a biological fluid, has attracted increasing attention in dermatology, pediatrics, toxicology, analytical chemistry, forensics, psychiatry, illicit drug screening, and infectious diseases [5, 6, 8, 12, 50]. The use of sweat in rapid diagnosis has an excellent potential for rapid biological analysis and health/disease surveillance [51]. Human sweat is clinically used to diagnose diseases such as Frey syndrome or panic disorder (PD), lactic acidosis, and CF in critical care patients. In some studies patients were screened for CF using sweat lactate. Furthermore, some medical centers use sweat to diagnose drug addiction and predict peripheral neuropathy occurrence in patients with diabetes [52, 53]. Sweat is also used to diagnose mental health conditions that cannot be diagnosed using laboratory analysis or auxiliary tests. PD is a type of paroxysmal neuropsychiatric disorder with an unknown etiology and pathogenesis. Hitherto, there is no reliable laboratory test for the diagnosis of PD. This may be due to the mechanism of discharge of neurotransmitter metabolites, which leads to an imbalance in sweat composition. In a study of 10 patients with active PD, there was no difference in blood parameter concentrations, although the concentrations of lactate, glucose, creatine, and magnesium in sweat were higher than those in patients with inactive PD [54]. In addition, the concentrations of lactate and other noninvasive skin metabolites was associated with cardiac insufficiency occurrence [55].

### Personal identification

Each individual is unique in terms of genes, lifestyle, and environment; thus, they express different biochemical compositions based on age, sex, level of activity, and other factors. Consequently, sweat metabolite concentrations vary from person to person. Without using DNA to distinguish individuals, in a model based on three compounds (lactate, urea, and glutamic acid), a bioaffinity detection system was used to determine the average absorbance change of each compound in sweat. Based on the analysis of natural sweat obtained from 25 people, it was proven that an enzymatic determination of the three analytes was effectively used to identify each person in two sample sets [56, 57]. This noninvasive analytical application would be beneficial in forensic science to verify personal identity through intelligent devices that monitor metabolites, as well as through the use of portable mass spectrometers combined with membrane probes to obtain volatile organic compounds released by metabolites in sweat [58].

Hemin, creatinine, phosphoric acid, uric acid, citric acid, and lactate were identified and used as potential biomarkers to distinguish between different kinds of body fluids. Jiang et al. used MALDI-MS and matrix *N*-(1-naphthyl) ethylenediamine dihydrochloride with high sensitivity and high salt tolerance to effectively detect some characteristic small metabolite molecules (such as hemin) in complex body fluid environments. The quantity of lactate in human body fluids may be measured in less than 10 min [12].

### Physiological monitoring of individual exercise and exercise analysis

This study represents a new point of interest in the scientific monitoring of kinematics and can stimulate the development of stand-alone portable systems related to sports [11, 59]. Once the equipment is placed on an athlete, the speed of motion, running frequency, knee-joint angle, and sweat lactate concentration can be monitored in real time [59]. This technology can attain different lactate duration limits and maximum lactate administration capacities; moreover, it can help athletes to develop a one-time workout plan.

### Noninvasive alternative to blood lactate analysis

It is important to replace invasive blood tests with noninvasive physiological biomarkers, to reduce the pain incurred by patients during invasive procedures and to obtain real-time human physiologic and pathological monitoring. Studies have evaluated the use of sensor technology, instead of invasive blood tests, to monitor the pharmacokinetics of levodopa and cannabinoids [6, 50, 60]. One of Yasu Kuno’s first hypotheses about sweat lactate was that sweat glands could secrete lactate into the blood during high-intensity exercise [9].

However, of the transport mechanisms of substances in sweat, only that for sodium and chloride is clear. The relationship between the concentrations of most sweat and blood components has hitherto not been established, with the exception of ethanol. Recent research has shown that most sweat components are independent of blood components, in terms of concentrations [1, 61].

Recent studies have shown a possible correlation between sweat and blood lactate concentrations. A study that monitored the physical fitness of athletes to determine the concentrations of lactate in sweat, venous blood, and capillary blood before and after maximum aerobic power exercise, found a correlation between an increase in blood and sweat lactate concentrations. Sweat lactate concentration is higher than blood lactate concentration and can be used to evaluate a change in the latter [61, 62]. Many studies have shown that the first measurement of lactate concentration in newly collected sweat is higher than later measurements, and may be related to the sweating rate; sweat lactate concentration decreases with increase in sweat volume [2, 15, 16, 61].

In contrast, some studies have shown that there is no correlation between sweat and blood lactate concentrations, suggesting that sweat lactate seems to originate from sweat glands independent of blood lactate. A study of 15 excellent amateur rugby players showed that blood and sweat concentrations were significantly positively correlated for urea and ammonia concentrations during official competitions, but not for lactate concentrations [63]. The noninvasive method may replace the method for determining lactate in the blood; however, further advances can only be made after further exploring the physiologic mechanism of sweat lactate production.

## Limitation and perspective on sweat lactate

In the past, medical treatment was administered to patients after clinical diagnosis resulting principally from physical examination. Analytes in biological fluids, such as blood and urine, could not be measured by physical examination; this represents a serious limitation of physical examination. In addition, it is difficult to detect analytes in urine, blood, and many body fluids using wearable sensors; this limited the development of remote health monitoring devices. According to Jason Heikenfeld, the first wave of technological development in the twentieth century triggered the development of analytical instruments for laboratory biological fluid testing, and the second wave of technology in the twenty-first century brought portable biofluid analysis instruments to the bedside of patients, so that doctors and nurses can quickly assess the physiologic status of patients [7]. The third wave of technology involves wearable transmission devices with a data display screen that allow patients to carry test results with them wherever they go and send them back remotely, thereby facilitating the remote real-time management of patients by medical personnel or health management agencies [64].

Recent studies have found that sweat lactate can be used in CF diagnosis, medication effect and disease monitoring, and identification of individuals [5, 6, 12]. Consequently, sweat lactate monitoring may provide information related to the physiological or pathological state of humans. However, sweat lactate concentration is affected by many factors, which limit its application. Future research should focus on how to ensure accurate sweat lactate monitoring, exclude the influence of variables, such as perspiration rate and evaporation, and eliminate interferences, such as temperature and humidity. Moreover, individual differences in sweat lactate concentration cannot be ignored. In addition to factors such as sweating amount, sweating rate, exercise intensity, local soft tissue hypoxia, and skin microorganisms, we believe that individual differences should be analyzed using proteomics to correct for some confounding factors. Furthermore, there remains a need to solve concrete problems the applicability of wearable sensors; for instance, the use of wearable sensors is limited by the patient’s position. The sacrum and coccyx are high-risk areas as far as the continuous measurement of lactate for PI prediction is concerned; this continuous measurement may increase the risk of PI occurrence. More so, multidisciplinary research is required for the development of soft wearable sensors, using materials that do not predispose the patient to PI occurrence. However, as mentioned above, there are many influencing factors of sweat production, and therefore, the relationship between sweat lactate concentration and soft tissue ischemia needs further exploration. The current research focuses on a small sample of healthy people in the laboratory [2, 15, 20]; hence, there is an urgent need for randomized clinical trials to verify the abovementioned relationship. Sweat lactate was used to assess the pathological status of the skin and soft tissues, and to clarify the relationship between sweat lactate concentration and PI occurrence. Moreover, even if these issues are resolved, real-time lactate monitoring will still be faced with the problem of the storage of data and records. Even with real-time monitoring, clinical staff also need manually recorded data. A corresponding real-time automatic input system should be developed to complete the task, as this will help the clinical staff to correct errors in the input data and improve work efficiency.

Based on current research on sweat, as a richly informative biofluid, we believe that sweat metabolites will be used in future to aid the medical assessment, prediction, detection, and treatment of conditions such as PI. Moreover, it will help in the follow-up of patients undergoing dialysis (who need dialysis monitoring and alert systems) and those with heart failure (who require alert systems). Sweat metabolites will also be useful in military combat effectiveness assessment, and the assessment of human tolerance in extreme environments.

## Conclusions

This review introduces the potential application of sweat lactate in bio-engineering. Based on the chemical properties of lactate and the physiological and biological responses of the skin, these detection techniques (lactate dehydrogenase measurement, chromatography, and electrochemical detection) provide the basis for predicting skin and soft tissue damage that may be caused by continuous work. While the previously used sweat analysis techniques are very sensitive and specific, it is still necessary to develop a fast and economical sensor technique. Further studies are required to ascertain whether sweat lactate can become a potential biomarker of human health, so as to provide strong basic theoretical support for subsequent commercial application. In addition, the wearable sensor for the detection of sweat lactate has good application prospects; however, no related research has compared the relationship between sweat and blood analysis results to determine the accuracy of wearable sensor monitoring of sweat lactate.

## Data Availability

All data generated or analyzed during this study are included in this published article.

## Abbreviations

CF: Cystic fibrosis
MALDI-MS: Matrix-assisted laser desorption/ionization mass spectrometry
PD: Panic disorder
PI: Pressure injury
